# Sense and non-sense of imaging in the era of organ preservation for rectal cancer

**DOI:** 10.1259/bjr.20230318

**Published:** 2023-09-26

**Authors:** Xinde Ou, Denise J van der Reijd, Doenja MJ Lambregts, Brechtje A Grotenhuis, Baukelien van Triest, Geerard L Beets, Regina GH Beets-Tan, Monique Maas

**Affiliations:** 1 Department of Radiology, The Netherlands Cancer Institute, Amsterdam, The Netherlands; 2 GROW School for Oncology and Developmental Biology, University of Maastricht, Maastricht, The Netherlands; 3 Department of Surgery, The Netherlands Cancer Institute, Amsterdam, The Netherlands; 4 Department of Radiation Oncology, The Netherlands Cancer Institute, Amsterdam, The Netherlands; 5 Institute of Regional Health Research, University of Southern Denmark, Odense, Denmark

## Abstract

This review summarizes the current applications and benefits of imaging modalities for organ preservation in the treatment of rectal cancer. The concept of organ preservation in the treatment of rectal cancer has revolutionized the way rectal cancer is managed. Initially, organ preservation was limited to patients with locally advanced rectal cancer who needed neoadjuvant therapy to reduce tumor size before surgery and achieved complete response. However, neoadjuvant therapy is now increasingly utilized for smaller and less aggressive tumors to achieve primary organ preservation. Additionally, more intensive neoadjuvant strategies are employed to improve complete response rates and increase the chances of successful organ preservation. The selection of patients for organ preservation is a critical component of treatment, and imaging techniques such as digital rectal exam, endoscopy, and MRI are commonly used for this purpose. In this review, we provide an overview of what imaging modalities should be chosen and how they can aid in the selection and follow-up of patients undergoing organ-preserving strategies.

## Introduction

About 20% of patients with rectal cancer who are treated with neoadjuvant chemoradiation achieve a complete response.^1^ Habr-Gama et al were the pioneers who first evaluated the possibility of not operating whilst carefully watching patients without residual tumor at endoscopy after chemoradiation.^2^ Although initially regarded with some suspicion and reluctance, this watchful waiting approach was further studied and became a successful alternative to TME.^3–5^ Currently, organ preservation is incorporated as a valid treatment option in complete responders to neoadjuvant chemoradiation into international guidelines.^6,7^

The possibility of organ preservation in rectal cancer has led to a paradigm shift in the treatment of rectal cancer. Initially, only patients with locally advanced rectal cancer who needed neoadjuvant treatment to obtain downsizing for a complete resection, and achieved a complete response were considered for organ preservation (referred to as secondary organ preservation). Nowadays, in pursuit of organ preservation, neoadjuvant treatment is also increasingly considered for smaller and lower risk tumors on the one hand (referred to as primary organ preservation), while on the other hand, more intensive neoadjuvant strategies are employed to achieve higher complete response rates to increase chances for organ preservation.^8^

Imaging plays a vital role in the selection of patients for organ preservation. The currently recommended triad of response evaluation is: digital rectal exam, endoscopy and MRI. The current review provides an overview on how to use the available imaging modalities in the selection for and follow-up of patients in organ preserving strategies.

## Imaging modalities in the field of organ preservation

The modalities that are available for response evaluation are: digital rectal exam (DRE or the ‘finger’), endoscopy (±biopsy), MRI, CT, endorectal ultrasound and fluorodeoxyglucose (FDG)-positron emission tomography (PET)-CT.

The combination of DRE and endoscopy is considered as the most reliable imaging examination for response assessment.^9–12^ MRI is also routinely recommended, because it provides complementary information to endoscopy on the former tumor bed that helps in distinguishing between residual tumor and treatment induced fibrosis, but also provides visualization of the entire pelvis (including the nodal basins).^13,14^ The combination of DRE, endoscopy, and MRI predicts absence of tumor (if all modalities indicate a CR) with a reported positive-predictive value of 98%.^9^

**Figure 1.**
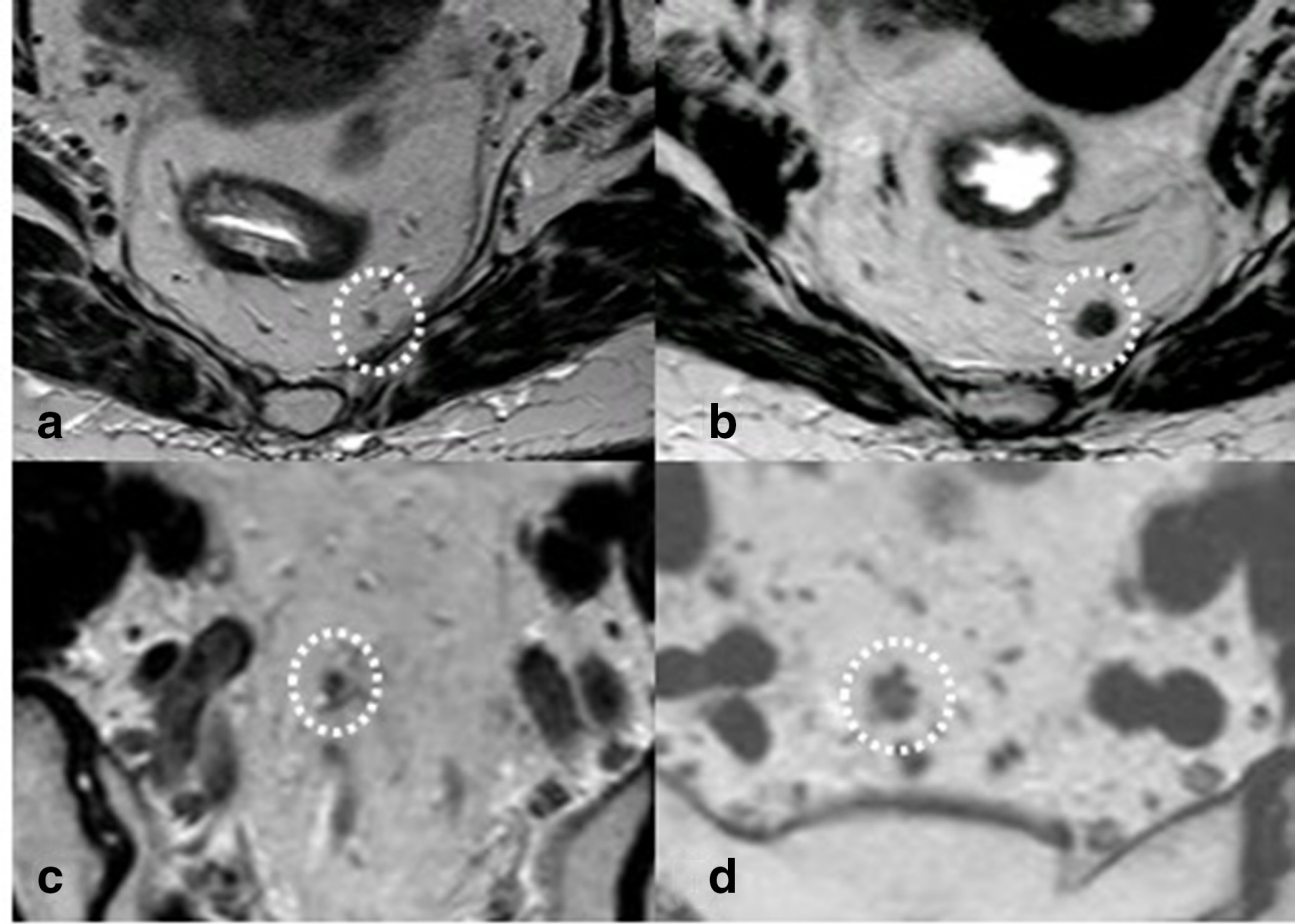
Two patients with nodal regrowths. Patient 1: in (a), the restaging post-CRT T2-weighted image is shown of a node that was malignant but became normalized after treatment. In (b), evident growth is visible. Patient 2: small residual node after CRT in (c) which grows and becomes morphologically suspect during follow-up (**d**). CRT, chemoradiotherapy.

**Figure 2.**
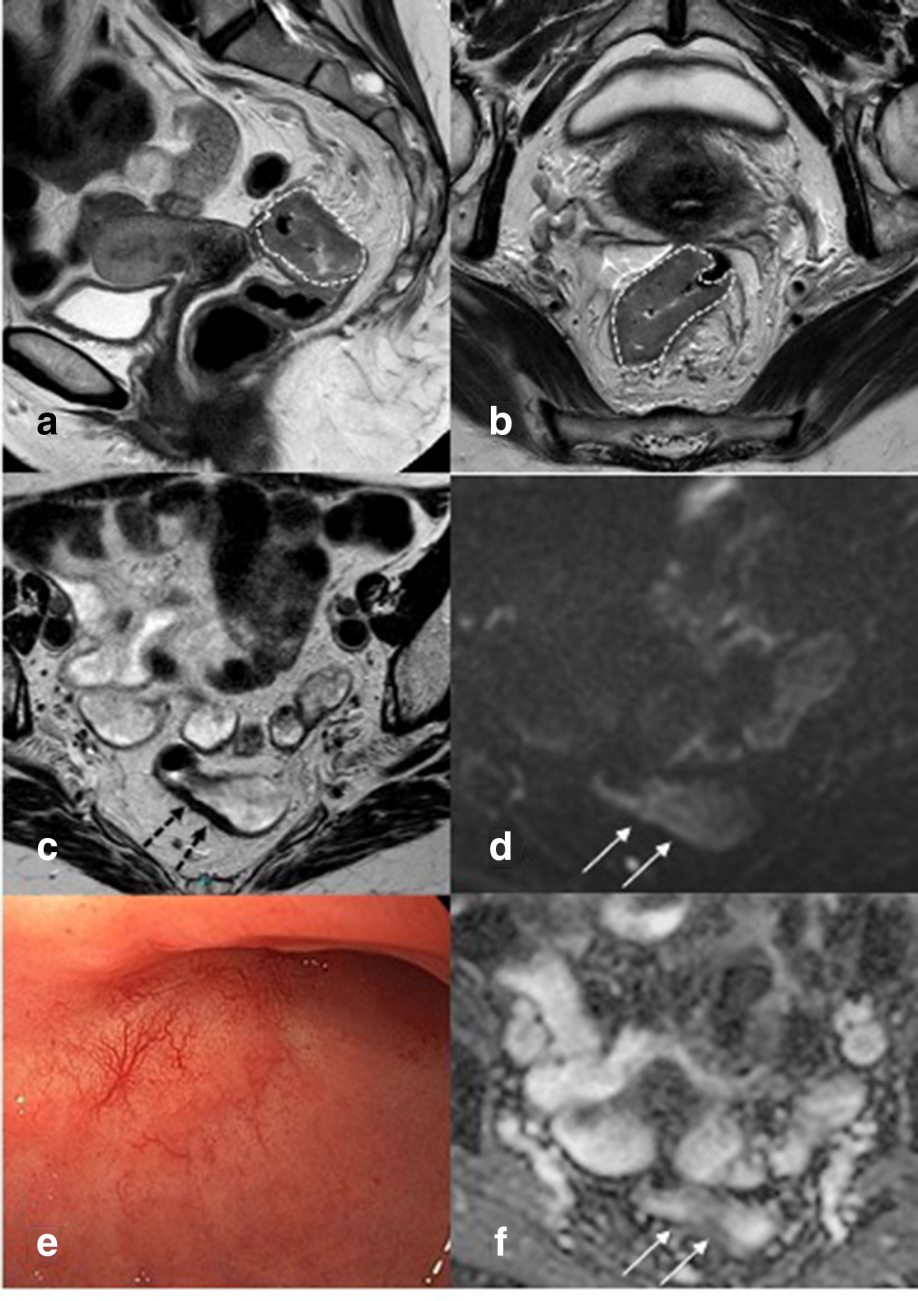
Complete response. Patient with a proximal rectal tumour with extramural growth on outlined with a dotted line on T2-weighted imaging on a and b before treatment. After chemoradiation (**c, d, f**) on the T2-weighted axial image (c, arrows) homogeneous fibrosis is visible at the former tumor location. At diffusion-weighted imaging, no residual restriction (**d, f**) is found. Endoscopy shows the typical image of a complete response: a white area with teleangiectasia (**e**).

**Figure 3.**
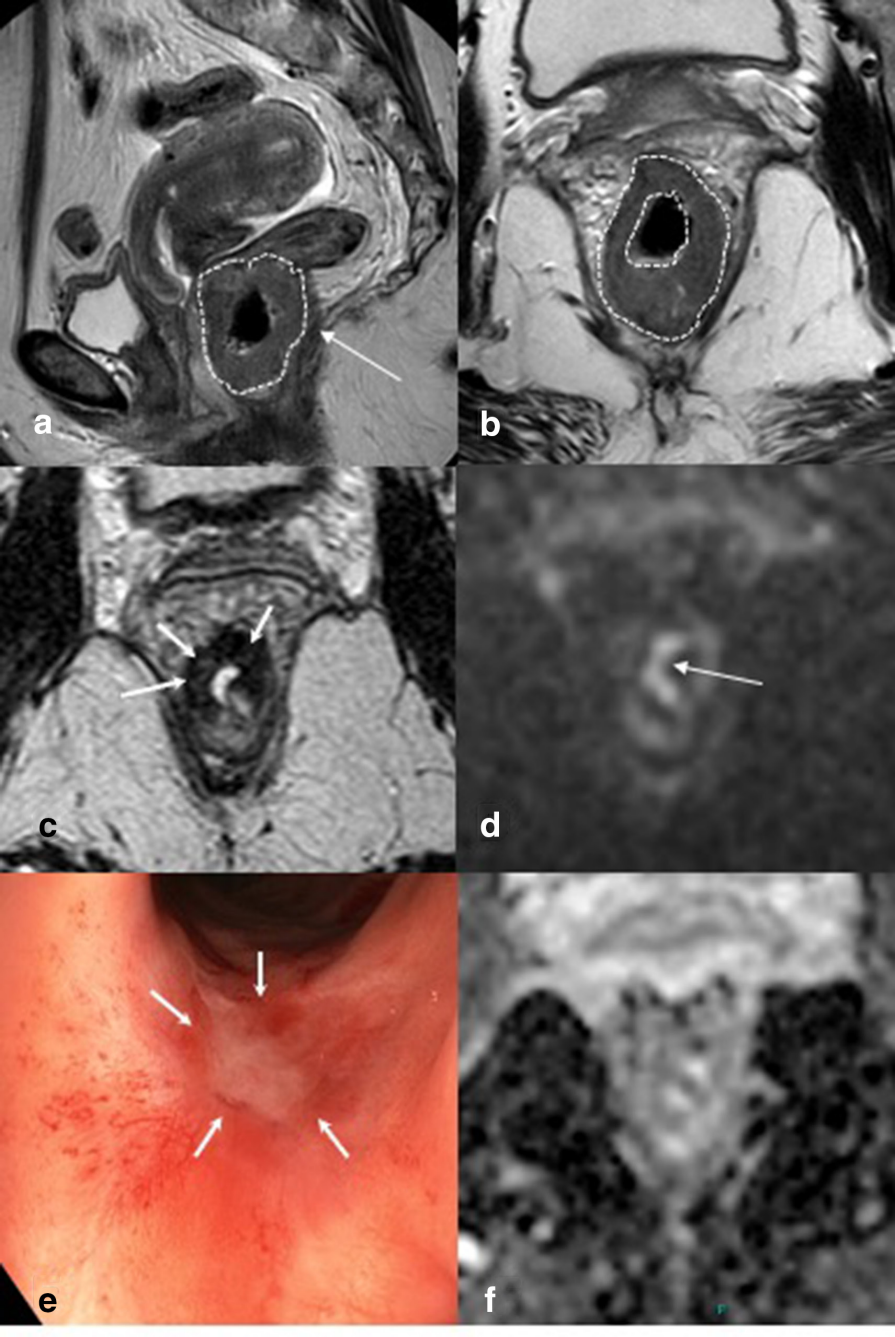
Near complete response. (a/b) Distal tumour outlined with dotted line with extramural extension on sagittal and axial T2-weighted imaging with invasion into the levator muscle (arrow in a). (**c**) After chemoradiation on axial T2-weighted imaging, a full thickness fibrosis is found with heterogeneous signal (white arrows in c). Endoscopy (**e**) shows an almost completely healed scar with central depression and a flat ulcer <1 cm (white arrows), surrounded by teleangiectasia. On DWI and ADC map (d/f) no restriction is found, only luminal T2 shine through (white arrow). ADC, apparent diffusion coefficient; DWI, diffusion-weighted imaging.

**Figure 4.**
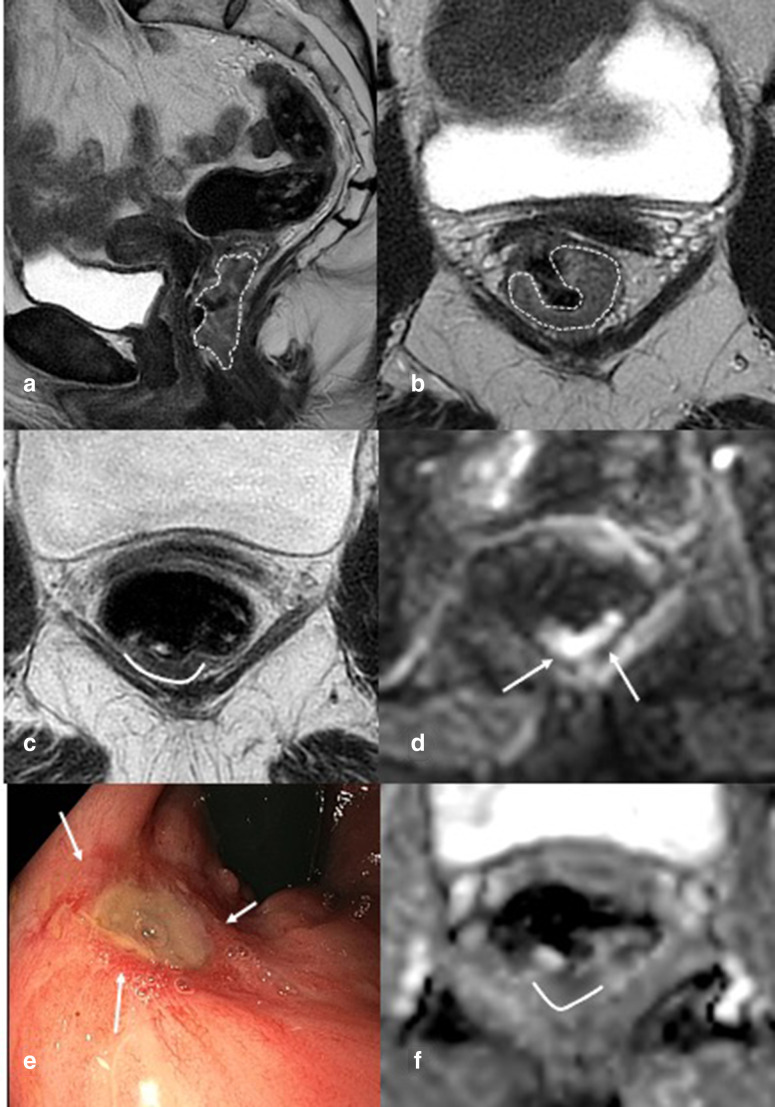
Small remnant. In (a/b), sagittal- and axial-weighted images of distal semi-circular tumor (outlined with dotted line), invading the anal canal. (c) Shows an axial T2-weighted image 12 weeks after chemoradiation. A small quite homogeneous wall thickening is visible with intermediate signal (outlined dorsally with a white line) and diffusion restriction (white arrows in d and line on ADC map in f). On endoscopy (**e**), an ulcer (deeper than the ulcer in Figure 3) with irregular edges is found (white arrows) and with DRE an irregular border is palpable. DRE, digital rectal exam.

Figures 1–4 show representative images of different responses on endoscopy and MRI.

### DRE and endoscopy

The main finding at DRE in patients with a clinical complete response (cCR) is absence of tumor. Usually, an entirely normal examination or slight rigidness of the area harboring the scar can be palpated. The presence of any detectable ulceration, irregularity, stenosis or mass is suspicious for residual tumor.^15^

The first described definition of a cCR on endoscopy by Habr-Gama et al was whitening of the mucosa or teleangiectasia with mucosal integrity. Habr-Gama et al only considered these patients for a non-operative approach.^15^ Currently, the ideal endoscopic finding of a cCR is the presence of only a white scar with or without slight teleangiectatic changes. The presence of ulcers, even superficial/shallow ulcers or significant areas of irregularity, does not qualify as typical CR. Particularly, large ulcers with irregular edges should be considered suspicious. Even though a typical white scar is considered indicative of a cCR, its positive-predictive value is 70–80%.^16^ So, in a subset of patients, residual tumor is missed. In these patients, small residual tumor islands are left in the scar, but not visible at endoscopy or MRI. Several studies have shown that tumors tend to regress from the inside towards the aggressive front, which may lead to only submucosal residual disease, which can explain the sometimes false-positive findings by using endoscopy only.^17,18^ In these cases, residual tumor could be only visible on MRI.

The other end of the spectrum also regularly occurs: in case of endoscopic abnormalities like adenomatous tissue or ulcers, subsequent surgery shows a pathologic complete response in up 89%.^19,20^ For these reasons, biopsy sampling is not routinely recommended for patients with a good response after neoadjuvant therapy. A negative-predictive value of only 11% has been reported by Perez et al, so false-negative results are very common.^9,21,22^

### MRI

Due to the high contrast resolution and field of view of MRI, it is the preferred imaging modality for response evaluation.

#### Protocol and patient preparation

For response evaluation after neoadjuvant therapy, the optimal protocol consists of three T2-weighted series (in sagittal, axial and coronal plane) with a slice thickness ≤3 mm, a diffusion-weighted sequence (with highest b-value ≥b800) and a large field of view T1 of T2 sequence from including the anal canal and reaching up to the promontory to ensure adequate depiction of the nodal stations. The use of a diffusion-weighted sequence for restaging is mandatory contrary to the primary staging setting, where it is optional.

The axial T2-weighted and diffusion-weighted sequence should be angled in the same way: perpendicular to the (former) tumor axis.^14,23^ This is necessary in order to allow for accurate anatomical localization of the signal (*i.e.* is the diffusion restriction located in the (former) tumor bed?). It is highly recommended to always compare with the pre-treatment images in order to accurately identify the tumor bed, as sometimes the relations between pelvic structures and tumor change due to the response.

There is no need for patient preparation or anti-spasmolytics. Use of a microenema to reduce gas in the rectum is recommended and has been shown to improve image quality on the diffusion-weighted sequence.^24^

#### Luminal response on MRI

##### Accuracy of MRI for response evaluation

Even though MRI is the mainstay of response evaluation, its diagnostic performance is far from perfect. Almost all patients develop fibrosis due to the radiation and it is very difficult to distinguish small areas of residual tumor within the fibrosis. For visual evaluation of T2W-MRI sensitivities to detect a pathological complete response (pCR) have been reported to range from 15 to 55%.^25–27^ Ruling out a pCR is much easier, specificities are reported to be >90%. Adding diffusion-MRI helps in increasing the sensitivity up to 84%, while maintaining a high specificity.^28^ The diffusion-weighted sequence will show high signal in dense tissues like tumor. The apparent diffusion coefficient (ADC) map quantifies the amount of diffusion and only in case of coinciding low ADC with the high signal on high b-value images there is true diffusion restriction.

##### Approaches to luminal response evaluation

Several methods have been proposed to assess response on MRI. Several groups have explored volumetry, both on T2W-MRI and diffusion-MRI. Volumetry does lead to very good results (up to 93% accuracy for post-CRT volume on diffusion).^29^ However, Sathyakumar et al showed that the radiologist’s visual evaluation of the diffusion-MRI was the best predictor for ypT0, even slightly outperforming DWI-volumetry (AUC 0.88 *vs* 0.84, respectively). Given the labor-intensiveness of volumetry, visual inspection is the preferred method to assess response.

The mrTRG system was introduced by Sclafani et al.^27^ The mrTRG represents a classification for T2W-MRI based on the pathologic TRG score by Mandard et al. The first report showed promising results, but a recent meta-analysis showed a sensitivity to detect a pCR of only 32%.^30^ This low sensitivity is similar to the low sensitivities found for visual evaluation of T2-weighted images and can be explained by the challenge to discern fibrosis from residual tumor. Later, a modified mrTRG system was introduced by Lee et al in which three categories were constructed based on T2-weighted images (complete/intermediate/poor regression, for which criteria were derived from the original mrTRG) and diffusion-weighted imaging (DWI).^31^ This modified 3-point mrTRG yielded a higher diagnostic performance than the original 5-point mrTRG when comparing with pTRG as the reference standard.

Santiago et al suggested to use the split scar sign to detect complete responders at T2W-MRI. In complete responders, the fibrotic wall thickening was described to show a layered aspect with regularly shaped layers of different signal intensity, while patients with residual tumor do not show a layered aspect or have interrupted layering. The sensitivity is slightly higher than the mrTRG (52%) but still leads to missing complete responders.^32^

A pattern-based approach incorporating pre-treatment morphology of the tumor, T2-signal intensity and shape after CRT as well as diffusion findings, showed a very good performance in the initial report (sensitivity of 94%, overall accuracy 88%). A later multicenter multireader validation showed a substantially lower sensitivity of 31–43%. The authors hypothesized that false-positive diffusion signal can be confusing, leading to overstaging residual tumor. In this same study, the pattern-based approach was compared to the 5-point mrTRG, 3-point modified mrTRG and split scar sign. The methods including DWI (pattern-based approach and modified mrTRG) outperformed the other methods, but had low sensitivities. Interobserver reproducibility was highest for the DWI pattern approach and lowest for the split scar sign (0.48 *vs* 0.18, respectively). Readers found the methods that included DWI the easiest to apply.^33^

### Nodal response

Nodal staging is challenging and to date no method has been proven to be accurate for nodal staging, regardless of many attempts to increase accuracy. In nodal staging both under- and overstaging occur, but after neoadjuvant therapy overstaging is encountered more frequently. MRI is the modality of choice for nodal staging. The ESGAR guideline for imaging of rectal cancer advises to use only size as a criterion, with a cut-off of 5 mm for the short-axis diameter and does not advocate the use of any morphologic features. However, if we use the 5 mm short axis cut-off after CRT, we overstage 24% and understage 18% of the cases.^34^ The vast majority of nodes will decrease in size after chemoradiation (the benign and the malignant ones). Suspicious nodes that show highly malignant morphological features before neoadjuvant treatment tend to decrease in size and become irregular fibrotic nodes with low signal on T2 and spicular margins. These nodes pose a particular problem to restage given their morphology.

One thing to keep in mind after CRT is that the likelihood for residual nodal involvement is lower than in a primary staging setting. However, a recent pooled analysis shows that ypN+ occurs in 7% of patients with ypT0.^35^

Lateral node involvement after CRT is even more challenging, as studies which correlate radiological findings with pathology data are lacking. Also, contrary to mesorectal nodes, lateral nodes disappear less frequently after CRT (44% *vs* 7–13%).^36–38^ Several cut-offs after CRT have been proposed for the short-axis of lateral nodes, but none has been validated yet.^39–43^ Morphologic criteria do not improve accuracy for restaging of lateral lymph nodes. The estimated incidence of residual lateral node involvement is 8–9%.^39,40^

Figure 1 shows two examples of nodal regrowth.

### Endorectal ultrasound (ERUS)

Some centres use ERUS for response evaluation, however it has not been adopted widely. ERUS, like MRI, has difficulty in distinguishing fibrosis from tumor, leading to low accuracy to detect pCR after CRT of 0–37%.^44–47^ Additionally, the efficacy is limited due to limited field of vue.^48–51^ Besides, it is highly operator-dependent and cannot be used in stenotic and proximal tumors.^52,53^ Therefore, ERUS is not recommended for post-CRT response evaluation, but can be a complementary modality in centers with ERUS-expertise.

### PET/CT

^18^F-FDG PET/CT has mainly been evaluated for its predictive value for a CR based on PET-parameters before and/or during CRT rather than its ability to detect CR based on post-therapy PET/CT.^54^ A review on the post-CRT diagnostic performance showed a positive-predictive value for pCR of 35% for maximum post-SUVmax.^55^ This can be explained by the low resolution of FDG-PET/CT to detect tumor sizes <5 mm. If FDG-PET/CT is used, its major strength is the identification of non-responders. However, FDG PET/CT is not accurate enough to select patients for organ preservation strategies.^55–57^ Furthermore, radionuclide techniques have limitations, such as the high cost and radiation. In general, it is not practically used for organ preservation, but in some selected cases, FDG-PET/CT can be considered as a problem solver. For example, to assess a questionable residual lateral node that does show response, but remains rather large and more information is needed before deciding on organ preservation.

### CT

CT lacks the inherent soft tissue resolution for discriminating rectal wall layers and involvement of the pelvic floor structures, and its use is confined to determine the presence of distant metastases.^58–60^

## Response evaluation: principles and treatment implications

Before the pioneering paper of Habr-Gama et al, response evaluation was not routine practice in rectal cancer treatment. When organ preservation/watchful waiting became a valid option for a cCR, response evaluation became routine and now is considered mandatory, since it has a major impact on treatment planning (with regard to organ preservation, but also surgical planning).^14^

### Selection for watchful waiting

In the first decade of watchful waiting, patients only with a typical cCR were considered for watchful waiting. A typical cCR was defined as absence of any residual abnormalities on DRE, endoscopy and MRI (Figure 2). However, by use of these criteria still up to 30% of patients with a complete response were not identified and denied organ preservation. Van der Sande et al evaluated patients with a pathological CR that were not identified at restaging and found that 84% showed luminal abnormalities at restaging before surgery. Apparently, the presence of these features is not always related to residual tumor and can lead to overstaging of the residual tumor and thus missing the opportunity of organ preservation in patients who actually have a complete response. These data led to the use of less strict selection criteria, where some minor residual abnormalities were accepted to allow for a longer waiting interval. Up to 90% of these patients eventually developed a typical cCR within 6 months after ending the chemoradiation.^61^ These patients are usually referred to as near complete responders (Figure 3). With regard to the definition of a near CR, a wide variety of terms and features are used and reliable evidence is lacking to support a uniform and reproducible definition.^62^ The wide spectrum of findings in a near CR ranges from small ulcers to small tumor remnants on endoscopy and from irregular fibrosis and minor residual signal on diffusion-MRI to heterogeneous circular fibrosis with multiple areas of punctate diffusion restriction.^62^ One can imagine that this wide spectrum leads to a high variation of outcome in these so-called near complete responders. In the absence of convincing evidence for any feature combination, selecting patients with a near CR for watchful waiting should be done with caution.

In summary, detection of a CR remains a challenge. These findings underline the need for integrated response evaluation by combining DRE, endoscopy and MRI (including DWI) in order to gain as much information as needed to establish response as accurately as possible.^9^

### Organ preservation with local treatment

In case of a small tumor remnant (<3 cm) on imaging after neoadjuvant therapy, organ preservation can still be pursued: a local treatment can be considered instead of TME, *e.g*. local excision^63–65^ or contact X-ray brachytherapy.^66^ This category of patients can be more easily identified as they show a residual mass on endoscopy, which can be measured on both endoscopy and MRI (Figure 4). In some cases, local excision or contact X-ray brachytherapy is also applied for near complete responders, depending on patient’s and local physician’s preference.

### Nodal response evaluation

For all organ preserving strategies, a prerequisite is the absence of malignant nodes after neoadjuvant therapy. Van der Sande et al showed in a series of missed complete responders that 25% was denied organ preservation due to residual irregular fibrotic nodes, that were erroneously deemed to be malignant.^19^ The ESGAR guideline on rectal cancer staging advises to consider nodes <5 mm after neoadjuvant therapy to be considered benign. However, in nodes >5 mm false-positive nodes are found. So, in case of a luminal complete response, nowadays a more flexible approach can be applied: when the nodes have responded, sometimes watchful waiting is considered, with close follow-up of the nodes. This approach is further supported by the low risk of nodal involvement in ypT0 of 7%.^35^ However, it should be considered that likelihood for nodal regrowth is higher than in patients with nodes <5 mm.

### Treatment in case of incomplete response

**Figure 5.**
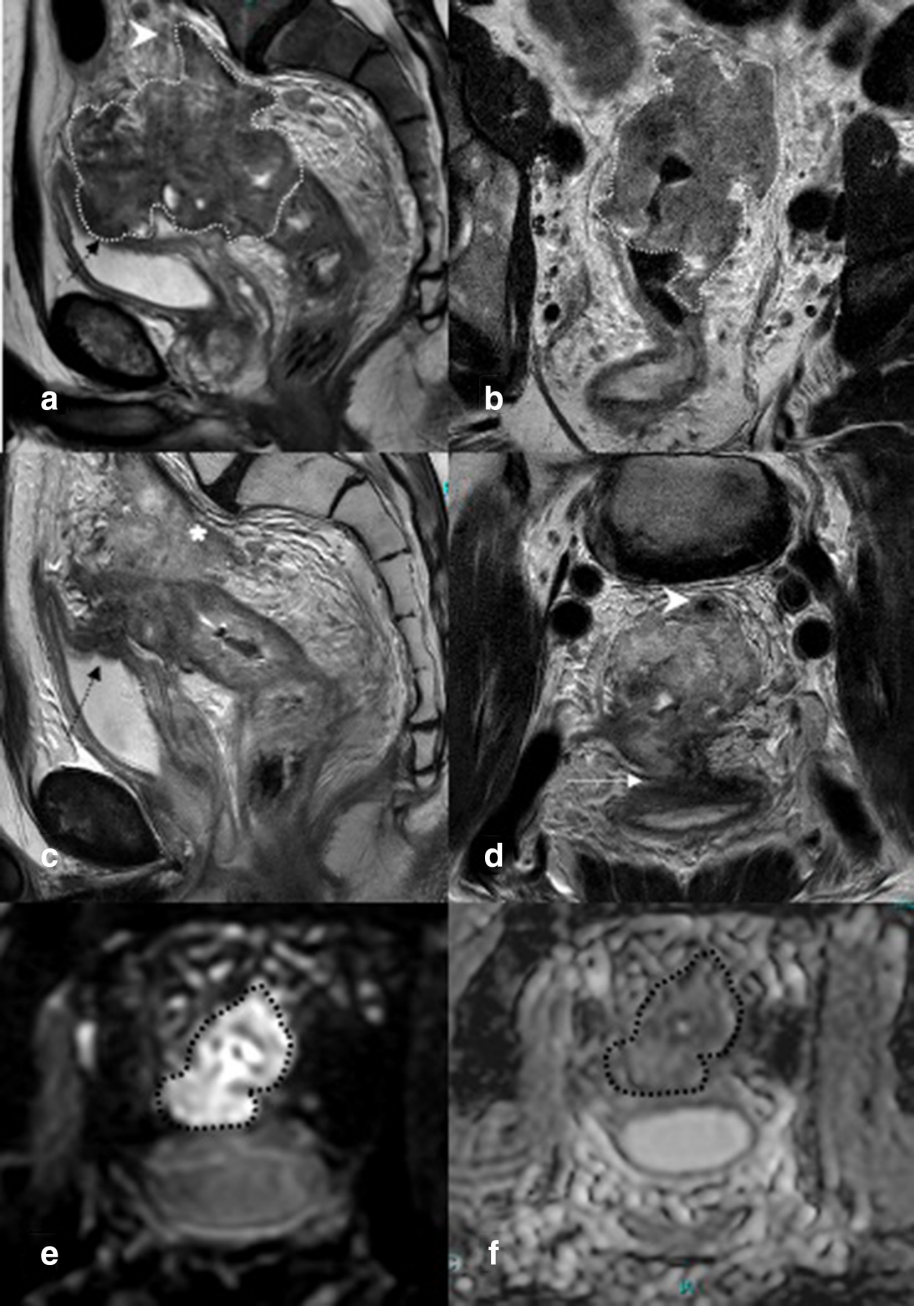
In (a and b), the primary tumor is delineated with a dotted line: a proximal rectal cancer massive extramural growth with EMVI (arrowhead in a) and invasion of the bladder (black arrow in a). After CRT on T2-weighted images (**c, d**), there is a decrease in volume, but obvious residual tumor is visible, still invading the bladder (arrow in d) and pre-sacral vessels (persistent EMVI, asterisk in c and arrowhead in d). The diffusion-weighted images (e/f) show obvious residual restriction (outlined with a black dotted line), confirming the residual tumor. Endoscopy is not necessary in these cases. EMVI, extramural venous invasion.

In case of obvious residual extramural disease (*e.g.* extramural extension of tumor with tumor signal, like extramural venous invasion (EMVI) or ycT3/4a/4b component) (Figure 5), obvious residual nodes or tumor deposits a TME is necessary to offer the patient a curative treatment. In these cases, the residual tumor needs to be restaged according to the TN(M) system to guide surgical planning.^14^

Table 1 summarizes the features associated with the different response groups

**Table 1.** Shows features associated with the different response groups.

Response category	DRE	Endoscopy	T2-weighted MRI	Diffusion-weighted MRI
cCR	No palpable abnormalities.	White scar ± teleangiectasia, if available: negative biopsy.	Normalized rectal wall, homogeneous regular (usually semicircular or focal) fibrosis, no suspicious lymph nodes or other extramural findings.	No diffusion restriction.
Near cCR or small remnant^*a*^	Small soft irregularity or ulcer, small residual mass.	Flat ulcer, mild persisting erythema of the scarflat ulcer, large ulcerlarge ulcer with irregular edges, adenomatous or granulomatous tissue,small residual mass (<3 cm), if available: negative, adenoma or carcinoma at biopsy.	Heterogeneous fibrosis, irregular fibrosis, mucinous spots in the fibrosis, regression of lymph nodes, no suspicious lymph nodes or other extramural findings.	Punctate diffusion restriction, linear diffusion restriction, multifocal diffusion restriction, mass like diffusion restriction (in case of small remnant).
Poor response	Palpable tumor mass.	Visible macroscopic tumor.	Residual tumor mass, persisting extramural disease. (*i.e.* nodes, tumor deposits, extamural invasion into the mesorectal fat, EMVI, MRF, PR and or other organs).	Mass-like diffusion restriction.

EMVI, extramural venous invasion; MRF, mesorectal fascia; cCR, clinical complete response.

a Given the lack of evidence to discern the near CR group from small tumor remnant, these categories were grouped and all features for both groups are mentioned.

#### Practical approach to response evaluation

Given the considerations above, a practical approach can be followed in response evaluation in order to answer the most important questions for the MDT and treatment decision-making. Haak et al proposed to use the MRI as a first step to assess the degree of response in order to guide the need for endoscopy and identify patients who can be selected for surgery immediately.^67^ To this end on T2-weighted and diffusion-weighted MRI patients with obvious poor response (defined as residual tumor on T2-weighted MRI and mass-like diffusion restriction) are distinguished from patients with at least good enough response to further evaluate with endoscopy. The patients with obvious residual disease can be restaged and discussed on the MDT to determine the surgical strategy. All patients allocated to the poor response group by the seven readers in the paper of Haak et al were histologically confirmed as having residual tumor, of which 73% had ypT3-4 tumor.^67^

For the patients with a good response, it is vital to qualify the response as: (1) clinically complete, (2) clinically near complete or (3) good, but small residual tumor, as this is used to guide the treatment decision-making. This categorization is based on DRE, endoscopy and MRI (T2W + DWI).

**Figure 6.**
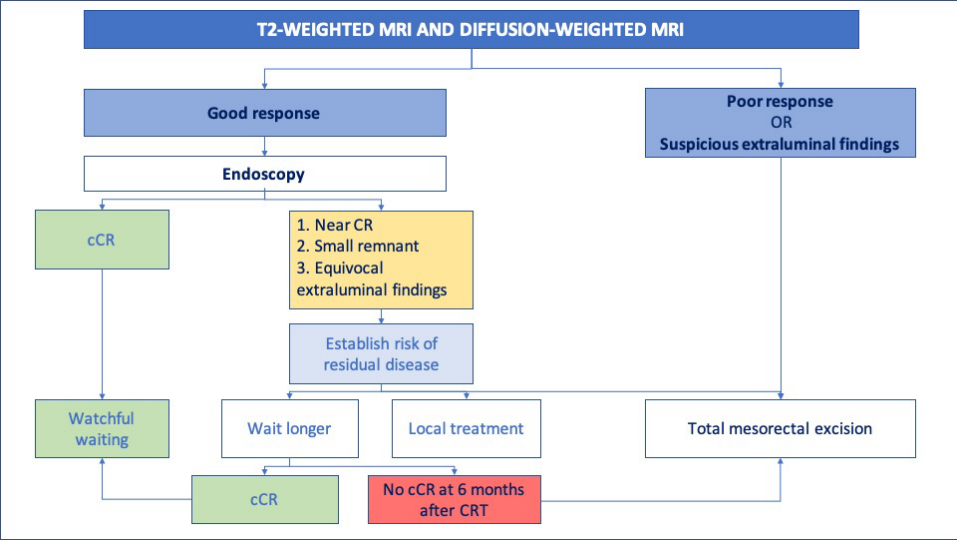
Practical approach to response evaluation with imaging. First response evaluation is recommended at 6–8 weeks after the last radiation fraction to avoid missing poor responders. In case of good response, another 6 week waiting interval can be applied. If at 12 weeks a cCR is found, patients can be referred for watchful waiting. In case of a near CR, the waiting interval can be further extended, but should be maximized to no longer than 6 months after the last radiation fraction. cCR, clinical complete response; nCR, near complete response; yMRF, mesorectal fascia status after chemoradiation; yEMVI, extramural venous invasion status after chemoradiation.

Figure 6 shows this approach in a flow diagram.

## Pitfalls in response evaluation

Sometimes post-radiation changes on T2-weighted images and DWI can mimic tumor throughout the rectum. If the former tumor location is not taken into account during response evaluation, these areas can be misinterpreted for tumor. Critical evaluation of the location of abnormal signal in the post-CRT images compared to the primary tumor is crucial to avoid these mistakes.

Tricky cases are patients who present with mucinous areas within the fibrosis. The current assumption is that if the tumor was not mucinous before the treatment, these areas likely represent pools of acellular mucin. In patients that had a primary mucinous tumor, it is very difficult to distinguish cellular from acellular mucin, especially since mucinous components usually show T2 shine through on diffusion series.^13^ In these cases, presence of residual cellular mucin cannot be excluded.

The diffusion-weighted sequence can be challenging to interpret and several pitfalls have been described. First, low signal on the ADC map is sometimes misinterpreted as suspicious residual tumor. However, this is only the case when it is accompanied with high signal on the b800/b1000 diffusion images. Dense fibrosis in itself (as often occurs after chemoradiation) gives low ADC signal, but will not show high signal on the high b-value diffusion images. Another pitfall is the differentiation of T2 shine through from tumor signal on DWI. On the high b-value diffusion images, high signal needs to correspond to low ADC on the ADC map in order to be diffusion restriction. Especially structures with high fluid content (with a very long T2 relaxation times) will continue to show high signal on the high b-value diffusion images, but will also show a high ADC. T2 shine through will typically have a star shape (as it usually corresponds to luminal fluid and the lumen is often star-shaped), while tumor will likely have a nodular, tubular or U-shape.

Last, artifacts due to air bubbles lead to high signal on DWI and can be misinterpreted as tumor. In these cases, it is important to correlate the signal to the former tumor location and fibrosis and the presence of air. In somewhat larger artifacts, the shape (geometrical distortions) can also be helpful in distinguishing artifacts from real signal from tumor.^13,68^

## Timing of imaging

Tumor response to neoadjuvant therapy is obviously time-dependent, and it has been suggested by multiple studies that the longer intervals used following the completion of the therapy, the higher rates of complete response, both clinical and pathological.^69,70^ Sloothaak et al reported that the highest chance of pCR would be reached in the 15th or 16th week after the start of CRT (10–11 weeks from the end of CRT),^69^ and there is even an increasing rate of cCR after ncCR with longer intervals than that.^71^ However, even when enough time is allowed for tumors to respond, not every patient will achieve a cCR. It is the fact that even with a longer wait, more than 70% of the patients without an initial cCR will not achieve a further response, while it can put them at risk of tumor progression or even metastasis.^72^ When looking at reports that evaluated the metabolic FDG uptake by the tumor uptake on PET/CT and estimates of tumor volume by MRI, it appears that the majority of tumor response after nCRT occurs within the first 6 weeks after RT completion.^72,73^ In order to avoid missing poor responders or even progression, it is advisable to perform a first response assessment quite early at 6–8 weeks after completion of CRT. In case of good response, an additional waiting interval can be added of 6 weeks to allow for further response. In case of a near CR at 12 weeks, again an additional waiting interval can be applied of 6–12 weeks to see how the response further evolves. However, it is advised to maximize the waiting time to 6 months, because a further response is unlikely when patients have not obtained a cCR at that time, and the risk for harming the patient increases.

## Imaging during follow-up

The main focus of follow-up is to detect local regrowths as early as possible, as this offers the patient the best chance for radical salvage surgery and good long-term outcome.^74^ From several studies, we know that local regrowths occur mainly within the first 12–18 months after entering the watch-and-wait protocol and that the vast majority of regrowths occur in the lumen (94%).^75^ Therefore, the emphasis of follow-up is on endoscopy. In the early era of organ preservation, 12-weekly endoscopy and CT were performed, but gradually longer intervals and MRI were implemented. Initially, in the first 2 years, a follow-up schedule was employed with a 3-monthly endoscopy and MRI, followed by 6-monthly imaging in year 3–5. Haak et al analyzed the local regrowths in the Dutch Watch-and-Wait cohort and the results led to adaptation of the FU schedule. Therefore, the current FU schedule recommended by the Dutch Watch-and-Wait consortium consists of endoscopies every 3 months during the first 2 years, accompanied by MRI in only the first year. During the second year, MRIs are recommended less often: every 6 months. In years 3–5, the number of MRIs can be further decreased to annual imaging, while endoscopy is maintained at a 6-monthly frequency (Table 2). This follow-up schedule better fits the actual time pattern of local regrowth occurrence. It should be noted, that in patients with residual tumor who were treated with local excision of contact X-ray brachytherapy, the risk for extramural recurrence is higher than in cCR treated with W&W. In these patients, more frequent MRI should be considered.

**Table 2.** Follow-up schedule as currently recommended by the Dutch Watch-and-Wait consortium

Follow-up time	Endoscopy	MRI
Year 1	3-monthly	3-monthly
Year 2	3-monthly	6-monthly
Year 3	6-monthly	Annual
Year 4	6-monthly	Annual
Year 5	6-monthly	Annual

When a local regrowth is found, it is confirmed by biopsy and an MRI is needed to restage the regrowth. As in primary staging and restaging after CRT, tumor height, tumor length, the T-stage, MRF-status, N-stage, EMVI and lateral node involvement should be described in order to guide surgery for the local regrowth. Last, but not least, a CT of chest and abdomen is required to search for distant metastases.

## Conclusion

Organ preservation has become part of routine treatment for patients with rectal cancer and given recent advances in treatment, it is expected that it will become increasingly popular, also driven by the rising patients’ interest. The mainstay of selection of patients for organ preservation is the triad of DRE, endoscopy and MRI. Other modalities lack sensitivity or resolution and some can be used as a problem solver. Radiologists play a pivotal role in pointing out the possibility of organ preservation based on their evaluation of the post-treatment MRI.

## References

[1] Maas M, Nelemans PJ, Valentini V, Das P, Rödel C, Kuo L-J, et al . Long-term outcome in patients with a pathological complete response after Chemoradiation for Rectal cancer: a pooled analysis of individual patient data. Lancet Oncol 2010; 11: 835–44. doi: 10.1016/S1470-2045(10)70172-8 20692872

[2] Habr-Gama A, de Souza PM, Ribeiro U, Nadalin W, Gansl R, Sousa AH, et al . Low Rectal cancer: impact of radiation and chemotherapy on surgical treatment. Dis Colon Rectum 1998; 41: 1087–96. doi: 10.1007/BF02239429 9749491

[3] Habr-Gama A, Perez RO, Nadalin W, Sabbaga J, Ribeiro U, Silva e Sousa AH, et al . Operative versus Nonoperative treatment for stage 0 distal Rectal cancer following Chemoradiation therapy: long-term results. Ann Surg 2004; 240: 711–18. doi: 10.1097/01.sla.0000141194.27992.32 15383798 PMC1356472

[4] van der Valk MJM, Hilling DE, Bastiaannet E, Meershoek-Klein Kranenbarg E, Beets GL, Figueiredo NL, et al . Long-term outcomes of clinical complete responders after Neoadjuvant treatment for Rectal cancer in the International watch & wait database (IWWD): an international Multicentre Registry study. Lancet 2018; 391: 2537–45. doi: 10.1016/S0140-6736(18)31078-X 29976470

[5] Maas M, Beets-Tan RGH, Lambregts DMJ, Lammering G, Nelemans PJ, Engelen SME, et al . Wait-and-see policy for clinical complete responders after Chemoradiation for Rectal cancer. J Clin Oncol 2011; 29: 4633–40. doi: 10.1200/JCO.2011.37.7176 22067400

[6] Benson AB, Venook AP, Al-Hawary MM, Azad N, Chen Y-J, Ciombor KK, et al . Rectal cancer, version 2.2022, NCCN clinical practice guidelines in oncology. J Natl Compr Canc Netw 2022; 20: 1139–67. doi: 10.6004/jnccn.2022.0051 36240850

[7] Glynne-Jones R, Wyrwicz L, Tiret E, Brown G, Rödel C, Cervantes A, et al . Rectal cancer: ESMO clinical practice guidelines for diagnosis, treatment and follow-up. Ann Oncol 2017; 28: iv22–40. doi: 10.1093/annonc/mdx224 28881920

[8] Garcia-Aguilar J, Patil S, Gollub MJ, Kim JK, Yuval JB, Thompson HM, et al . Organ preservation in patients with Rectal adenocarcinoma treated with total Neoadjuvant therapy. J Clin Oncol 2022; 40: 2546–56. doi: 10.1200/JCO.22.00032 35483010 PMC9362876

[9] Maas M, Lambregts DMJ, Nelemans PJ, Heijnen LA, Martens MH, Leijtens JWA, et al . Assessment of clinical complete response after chemoradiation for rectal cancer with digital rectal examination, Endoscopy, and MRI: selection for organ-saving treatment. Ann Surg Oncol 2015; 22: 3873–80. doi: 10.1245/s10434-015-4687-9 26198074 PMC4595525

[10] Fokas E, Appelt A, Glynne-Jones R, Beets G, Perez R, Garcia-Aguilar J, et al . International consensus recommendations on key outcome measures for organ preservation after (Chemo)Radiotherapy in patients with Rectal cancer. Nat Rev Clin Oncol 2021; 18: 805–16. doi: 10.1038/s41571-021-00538-5 34349247

[11] Felder SI, Feuerlein S, Parsee A, Imanirad I, Sanchez J, Dessureault S, et al . Endoscopic and MRI response evaluation following neoadjuvant treatment for rectal cancer: a pictorial review with matched MRI, endoscopic, and pathologic examples. Abdom Radiol (NY). 2021;46():1783-804. doi: 10.1007/s00261-020-02827-6. 33111189

[12] Kimura C, Crowder SE, Kin C. Is It Really Gone? Assessing Response to Neoadjuvant Therapy in Rectal Cancer. J Gastrointest Cancer. 2022. doi: 10.1007/s12029-022-00889-x. 36417142

[13] Lambregts DMJ, Boellaard TN, Beets-Tan RGH . Response evaluation after Neoadjuvant treatment for Rectal cancer using modern MR imaging: a pictorial review. Insights Imaging 2019; 10(): 15. doi: 10.1186/s13244-019-0706-x 30758688 PMC6375095

[14] Beets-Tan RGH, Lambregts DMJ, Maas M, Bipat S, Barbaro B, Curvo-Semedo L, et al . Correction to: magnetic resonance imaging for clinical management of Rectal cancer: updated recommendations from the 2016 European society of gastrointestinal and abdominal Radiology (ESGAR) consensus meeting. Eur Radiol 2018; 28(): 2711. doi: 10.1007/s00330-017-5204-2 29322331 PMC6828070

[15] Habr-Gama A, Perez RO, Wynn G, Marks J, Kessler H, Gama-Rodrigues J. Complete clinical response after neoadjuvant chemoradiation therapy for distal rectal cancer: characterization of clinical and endoscopic findings for standardization. Dis Colon Rectum. 2010;53():1692-8. doi: 10.1007/DCR.0b013e3181f42b89. 21178866

[16] van der Sande ME, Beets GL . Predictive value of endoscopic features for a complete response after Chemoradiotherapy for Rectal cancer Ann Surg 2021; 274: e724–25. doi: 10.1097/SLA.0000000000004012 32516235

[17] Duldulao MP, Lee W, Streja L, Chu P, Li W, Chen Z, et al . Distribution of residual cancer cells in the bowel wall after neoadjuvant chemoradiation in patients with rectal cancer. Dis Colon Rectum. 2013;56():142-9. doi: 10.1097/DCR.0b013e31827541e2. 23303141 PMC4674069

[18] Perez RO, Habr-Gama A, Smith FM, Kosinski L, Sao Juliao GP, Grzona E, et al . Fragmented pattern of tumor regression and lateral intramural spread may influence margin appropriateness after TEM for rectal cancer following neoadjuvant CRT. J Surg Oncol. 2014;109():853-8. doi: 10.1002/jso.23571. 24862927

[19] van der Sande ME, Beets GL, Hupkens BJ, Breukink SO, Melenhorst J, Bakers FC, et al . Response assessment after (Chemo)Radiotherapy for Rectal cancer: Why are we missing complete responses with MRI and Endoscopy? Eur J Surg Oncol 2019; 45: 1011–17. doi: 10.1016/j.ejso.2018.11.019 30528891

[20] Nahas SC, Rizkallah Nahas CS, Sparapan Marques CF, Ribeiro U Jr., Cotti GC, Imperiale AR, et al . Pathologic Complete Response in Rectal Cancer: Can We Detect It? Lessons Learned From a Proposed Randomized Trial of Watch-and-Wait Treatment of Rectal Cancer. Dis Colon Rectum. 2016;59():255-63. doi: 10.1097/DCR.0000000000000558. 26953983

[21] Perez RO, Habr-Gama A, Pereira GV, Lynn PB, Alves PA, Proscurshim I, et al . Role of biopsies in patients with residual Rectal cancer following Neoadjuvant Chemoradiation after downsizing: can they rule out persisting cancer Colorectal Dis 2012; 14: 714–20. doi: 10.1111/j.1463-1318.2011.02761.x 22568644

[22] Martens MH, Maas M, Heijnen LA, Lambregts DMJ, Leijtens JWA, Stassen LPS, et al . Long-term outcome of an organ preservation program after Neoadjuvant treatment for Rectal cancer. J Natl Cancer Inst 2016; 108: 12: djw171. doi: 10.1093/jnci/djw171 27509881

[23] Santiago I, Rodrigues B, Barata M, Figueiredo N, Fernandez L, Galzerano A, et al . “Re-staging and follow-up of Rectal cancer patients with MR imaging when "watch-and-wait" is an option: a practical guide”. Insights Imaging 2021; 12(): 114. doi: 10.1186/s13244-021-01055-w 34373961 PMC8353037

[24] van Griethuysen JJM, Bus EM, Hauptmann M, Lahaye MJ, Maas M, Ter Beek LC, et al . Gas-induced susceptibility Artefacts on diffusion-weighted MRI of the Rectum at 1.5 T - effect of applying a micro-Enema to improve image quality. Eur J Radiol 2018; 99: 131–37. doi: 10.1016/j.ejrad.2017.12.020 29362144

[25] Yuval JB, Patil S, Gangai N, Omer DM, Akselrod DG, Fung A, et al . MRI assessment of Rectal cancer response to Neoadjuvant therapy: a Multireader study. Eur Radiol 2023; 33: 5761–68. doi: 10.1007/s00330-023-09480-9 36814032 PMC10394731

[26] de Jong EA, ten Berge J, Dwarkasing RS, Rijkers AP, van Eijck CHJ . The accuracy of MRI, Endorectal Ultrasonography, and computed tomography in predicting the response of locally advanced Rectal cancer after preoperative therapy: a Metaanalysis. Surgery 2016; 159: 688–99. doi: 10.1016/j.surg.2015.10.019 26619929

[27] Sclafani F, Brown G, Cunningham D, Wotherspoon A, Mendes LST, Balyasnikova S, et al . Comparison between MRI and pathology in the assessment of tumour regression grade in Rectal cancer. Br J Cancer 2017; 117: 1478–85. doi: 10.1038/bjc.2017.320 28934761 PMC5680467

[28] van der Paardt MP, Zagers MB, Beets-Tan RGH, Stoker J, Bipat S . Patients who undergo preoperative Chemoradiotherapy for locally advanced Rectal cancer Restaged by using diagnostic MR imaging: a systematic review and meta-analysis. Radiology 2013; 269: 101–12. doi: 10.1148/radiol.13122833 23801777

[29] Curvo-Semedo L, Lambregts DMJ, Maas M, Thywissen T, Mehsen RT, Lammering G, et al . Rectal cancer: assessment of complete response to preoperative combined radiation therapy with chemotherapy--conventional MR Volumetry versus diffusion-weighted MR imaging. Radiology 2011; 260: 734–43. doi: 10.1148/radiol.11102467 21673229

[30] Jang JK, Choi SH, Park SH, Kim KW, Kim HJ, Lee JS, et al . MR tumor regression grade for pathological complete response in Rectal cancer post Neoadjuvant Chemoradiotherapy: a systematic review and meta-analysis for accuracy. Eur Radiol 2020; 30: 2312–23. doi: 10.1007/s00330-019-06565-2 31953656

[31] Lee MA, Cho SH, Seo AN, Kim HJ, Shin K-M, Kim SH, et al . Modified 3-point MRI-based tumor regression grade incorporating DWI for locally advanced Rectal cancer. AJR Am J Roentgenol 2017; 209: 1247–55. doi: 10.2214/AJR.16.17242 28981353

[32] Santiago I, Barata M, Figueiredo N, Parés O, Henriques V, Galzerano A, et al . The split scar sign as an indicator of sustained complete response after Neoadjuvant therapy in Rectal cancer. Eur Radiol 2020; 30: 224–38. doi: 10.1007/s00330-019-06348-9 31350587

[33] El Khababi N, Beets-Tan RGH, Tissier R, Lahaye MJ, Maas M, Curvo-Semedo L, et al . Comparison of MRI response evaluation methods in Rectal cancer: a Multicentre and Multireader validation study. Eur Radiol 2023; 33: 4367–77. doi: 10.1007/s00330-022-09342-w 36576549

[34] Pangarkar S, Mistry K, Choudhari A, Smriti V, Ahuja A, Katdare A, et al . Accuracy of MRI for nodal Restaging in Rectal cancer: a retrospective study of 166 cases. Abdom Radiol (NY) 2021; 46: 498–505. doi: 10.1007/s00261-020-02708-y 32813028

[35] Haak HE, Beets GL, Peeters K, Nelemans PJ, Valentini V, Rödel C, et al . Prevalence of nodal involvement in Rectal cancer after Chemoradiotherapy. Br J Surg 2021; 108: 1251–58. doi: 10.1093/bjs/znab194 34240110 PMC8604154

[36] Atef Y, Koedam TW, van Oostendorp SE, Bonjer HJ, Wijsmuller AR, Tuynman JB . Lateral pelvic lymph node metastases in Rectal cancer: A systematic review. World J Surg 2019; 43: 3198–3206. doi: 10.1007/s00268-019-05135-3 31468119

[37] Yamaoka Y, Kinugasa Y, Shiomi A, Yamaguchi T, Kagawa H, Yamakawa Y, et al . Preoperative Chemoradiotherapy changes the size criterion for predicting lateral lymph node metastasis in lower Rectal cancer. Int J Colorectal Dis 2017; 32: 1631–37. doi: 10.1007/s00384-017-2873-x 28762190

[38] Heijnen LA, Maas M, Beets-Tan RG, Berkhof M, Lambregts DM, Nelemans PJ, et al . Nodal staging in Rectal cancer: why is Restaging after Chemoradiation more accurate than primary nodal staging Int J Colorectal Dis 2016; 31: 1157–62. doi: 10.1007/s00384-016-2576-8 27055660 PMC4867151

[39] Kawai K, Shiratori H, Hata K, Nozawa H, Tanaka T, Nishikawa T, et al . Optimal size criteria for lateral lymph node dissection after Neoadjuvant Chemoradiotherapy for Rectal cancer. Dis Colon Rectum 2021; 64: 274–83. doi: 10.1097/DCR.0000000000001866 33395141

[40] Ishihara S, Kawai K, Tanaka T, Kiyomatsu T, Hata K, Nozawa H, et al . Oncological outcomes of lateral pelvic lymph node metastasis in Rectal cancer treated with preoperative Chemoradiotherapy. Dis Colon Rectum 2017; 60: 469–76. doi: 10.1097/DCR.0000000000000752 28383446

[41] Ogura A, Konishi T, Beets GL, Cunningham C, Garcia-Aguilar J, Iversen H, et al . Lateral nodal features on Restaging magnetic resonance imaging associated with lateral local recurrence in low Rectal cancer after Neoadjuvant Chemoradiotherapy or radiotherapy. JAMA Surg 2019; 154(): e192172. doi: 10.1001/jamasurg.2019.2172 31268504 PMC6613303

[42] Schaap DP, Boogerd LSF, Konishi T, Cunningham C, Ogura A, Garcia-Aguilar J, et al . Rectal cancer lateral lymph nodes: Multicentre study of the impact of Obturator and internal iliac nodes on Oncological outcomes. Br J Surg 2021; 108: 205–13. doi: 10.1093/bjs/znaa009 33711144

[43] Ogura A, van Oostendorp S, Kusters M . Neoadjuvant (Chemo)Radiotherapy and lateral node dissection: is it mutually exclusive Clin Colon Rectal Surg 2020; 33: 355–60. doi: 10.1055/s-0040-1714239 33162839 PMC7605909

[44] Huh JW, Park YA, Jung EJ, Lee KY, Sohn SK . Accuracy of Endorectal Ultrasonography and computed tomography for Restaging Rectal cancer after preoperative Chemoradiation. J Am Coll Surg 2008; 207: 7–12. doi: 10.1016/j.jamcollsurg.2008.01.002 18589355

[45] Zhao RS, Wang H, Zhou ZY, Zhou Q, Mulholland MW . Restaging of locally advanced Rectal cancer with magnetic resonance imaging and Endoluminal ultrasound after preoperative Chemoradiotherapy: a systemic review and meta-analysis. Dis Colon Rectum 2014; 57: 388–95. doi: 10.1097/DCR.0000000000000022 24509465

[46] Liu S, Zhong G-X, Zhou W-X, Xue H-D, Pan W-D, Xu L, et al . Can Endorectal ultrasound, MRI, and mucosa integrity accurately predict the complete response for mid-low Rectal cancer after preoperative Chemoradiation? A prospective observational study from a single medical center. Dis Colon Rectum 2018; 61: 903–10. doi: 10.1097/DCR.0000000000001135 29944579

[47] Chen L, Liu X, Zhang W, Qin S, Wang Y, Lin J, et al . The predictive value of tumor volume reduction ratio on three-dimensional Endorectal ultrasound for tumor response to Chemoradiotherapy for locally advanced Rectal cancer. Ann Transl Med 2022; 10: 666. doi: 10.21037/atm-22-2418 35845508 PMC9279805

[48] Kennedy E, Vella ET, Blair Macdonald D, Wong CS, McLeod R, Cancer Care Ontario Preoperative Assessment for Rectal Cancer Guideline Development Group . Optimisation of preoperative assessment in patients diagnosed with Rectal cancer. Clin Oncol (R Coll Radiol) 2015; 27: 225–45. doi: 10.1016/j.clon.2015.01.001 25656631

[49] Tudyka V, Blomqvist L, Beets-Tan RGH, Boelens PG, Valentini V, van de Velde CJ, et al . EURECCA consensus conference highlights about colon & Rectal cancer Multidisciplinary management: the Radiology experts review. Eur J Surg Oncol 2014; 40: 469–75. doi: 10.1016/j.ejso.2013.10.029 24439446

[50] Fowler KJ, Kaur H, Cash BD, Feig BW, Gage KL, Garcia EM, et al . ACR appropriateness Criteria((R)) pretreatment staging of colorectal cancer. Journal of the American College of Radiology 2017; 14: S234–44. doi: 10.1016/j.jacr.2017.02.012 28473079

[51] Heo SH, Kim JW, Shin SS, Jeong YY, Kang HK. Multimodal imaging evaluation in staging of rectal cancer. World J Gastroenterol. 2014;20():4244-55. doi: 10.3748/wjg.v20.i15.4244. 24764662 PMC3989960

[52] Akasu T, Kondo H, Moriya Y, Sugihara K, Gotoda T, Fujita S, et al . Endorectal ultrasonography and treatment of early stage rectal cancer. World J Surg. 2000;24():1061-8. doi: 10.1007/s002680010151. 11036283

[53] Brown G, Davies S, Williams GT, Bourne MW, Newcombe RG, Radcliffe AG, et al . Effectiveness of preoperative staging in Rectal cancer: Digital Rectal examination, Endoluminal ultrasound or magnetic resonance imaging Br J Cancer 2004; 91: 23–29. doi: 10.1038/sj.bjc.6601871 15188013 PMC2364763

[54] Maffione AM, Marzola MC, Capirci C, Colletti PM, Rubello D. Value of (18)F-FDG PET for Predicting Response to Neoadjuvant Therapy in Rectal Cancer: Systematic Review and Meta-Analysis. AJR Am J Roentgenol. 2015;204():1261-8. doi: 10.2214/AJR.14.13210. 26001237

[55] Joye I, Deroose CM, Vandecaveye V, Haustermans K . The role of diffusion-weighted MRI and (18)F-FDG PET/CT in the prediction of pathologic complete response after Radiochemotherapy for Rectal cancer: a systematic review. Radiother Oncol 2014; 113: 158–65. doi: 10.1016/j.radonc.2014.11.026 25483833

[56] Dos Anjos DA, Perez RO, Habr-Gama A, São Julião GP, Vailati BB, Fernandez LM, et al . Semiquantitative Volumetry by sequential PET/CT may improve prediction of complete response to Neoadjuvant Chemoradiation in patients with distal Rectal cancer. Dis Colon Rectum 2016; 59: 805–12. doi: 10.1097/DCR.0000000000000655 27505108

[57] Rymer B, Curtis NJ, Siddiqui MRS, Chand M . FDG PET/CT can assess the response of locally advanced Rectal cancer to Neoadjuvant Chemoradiotherapy: evidence from meta-analysis and systematic review. Clin Nucl Med 2016; 41: 371–75. doi: 10.1097/RLU.0000000000001166 26914561

[58] Mathur P, Smith JJ, Ramsey C, Owen M, Thorpe A, Karim S, et al . Comparison of CT and MRI in the pre-operative staging of Rectal adenocarcinoma and prediction of Circumferential resection margin involvement by MRI. Colorectal Dis 2003; 5: 396–401. doi: 10.1046/j.1463-1318.2003.00537.x 12925069

[59] Maizlin ZV, Brown JA, So G, Brown C, Phang TP, Walker ML, et al . Can CT replace MRI in preoperative assessment of the Circumferential resection margin in Rectal cancer Dis Colon Rectum 2010; 53: 308–14. doi: 10.1007/DCR.0b013e3181c5321e 20173478

[60] Vliegen R, Dresen R, Beets G, Daniels-Gooszen A, Kessels A, van Engelshoven J, et al . The accuracy of multi-detector row CT for the assessment of tumor invasion of the Mesorectal Fascia in primary Rectal cancer. Abdom Imaging 2008; 33: 604–10. doi: 10.1007/s00261-007-9341-y 18175167 PMC2491404

[61] Hupkens BJP, Maas M, Martens MH, van der Sande ME, Lambregts DMJ, Breukink SO, et al . Organ preservation in Rectal cancer after Chemoradiation: should we extend the observation period in patients with a clinical near-complete response Ann Surg Oncol 2018; 25: 197–203. doi: 10.1245/s10434-017-6213-8 29134378

[62] Custers PA, Geubels BM, Beets GL, Lambregts DMJ, van Leerdam ME, van Triest B, et al . Defining near-complete response following (Chemo)Radiotherapy for Rectal cancer: systematic review. Br J Surg 2022; 110: 43–49. doi: 10.1093/bjs/znac372 36349555

[63] Bao QR, Ferrari S, Capelli G, Ruffolo C, Scarpa M, Agnes A, et al . Rectal sparing approaches after Neoadjuvant treatment for Rectal cancer. A Systematic Review and Meta-Analysis Comparing Local Excision and Watch and Wait Cancers (Basel) 2023; 15(. doi: 10.3390/cancers15020465 PMC9856629 36672414

[64] Rullier E, Vendrely V, Asselineau J, Rouanet P, Tuech J-J, Valverde A, et al . Organ preservation with Chemoradiotherapy plus local Excision for Rectal cancer: 5-year results of the GRECCAR 2 randomised trial. The Lancet Gastroenterology & Hepatology 2020; 5: 465–74. doi: 10.1016/S2468-1253(19)30410-8 32043980

[65] Al-Najami I, Jones HJ, Dickson EA, Muirhead R, Deding U, James DR, et al . Rectal cancer: watch-and-wait and continuing the Rectal-preserving strategy with local Excision for incomplete response or limited regrowth. Surg Oncol 2021; 37: 101574. doi: 10.1016/j.suronc.2021.101574 33853031

[66] Custers PA, Maas M, Lambregts DMJ, Beets-Tan RGH, Beets GL, Peters FP, et al . Features on Endoscopy and MRI after treatment with contact X-ray Brachytherapy for Rectal cancer: Explorative results. Cancers (Basel) 2022; 14: 22: 5565. doi: 10.3390/cancers14225565 PMC9688812 36428659

[67] Haak HE, Maas M, Lahaye MJ, Boellaard TN, Delli Pizzi A, Mihl C, et al . Selection of patients for organ preservation after Chemoradiotherapy: MRI identifies poor responders who can go straight to surgery. Ann Surg Oncol 2020; 27: 2732–39. doi: 10.1245/s10434-020-08334-8 32172333

[68] Lambregts DMJ, van Heeswijk MM, Delli Pizzi A, van Elderen SGC, Andrade L, Peters NHGM, et al . Diffusion-weighted MRI to assess response to Chemoradiotherapy in Rectal cancer: main interpretation pitfalls and their use for teaching. Eur Radiol 2017; 27: 4445–54. doi: 10.1007/s00330-017-4830-z 28409357

[69] Sloothaak DAM, Geijsen DE, van Leersum NJ, Punt CJA, Buskens CJ, Bemelman WA, et al . Optimal time interval between Neoadjuvant Chemoradiotherapy and surgery for Rectal cancer. Br J Surg 2013; 100: 933–39. doi: 10.1002/bjs.9112 23536485

[70] Kalady MF, de Campos-Lobato LF, Stocchi L, Geisler DP, Dietz D, Lavery IC, et al . Predictive factors of pathologic complete response after Neoadjuvant Chemoradiation for Rectal cancer. Ann Surg 2009; 250: 582–89. doi: 10.1097/SLA.0b013e3181b91e63 19710605

[71] Gambacorta MA, Masciocchi C, Chiloiro G, Meldolesi E, Macchia G, van Soest J, et al . Timing to achieve the highest rate of pCR after preoperative Radiochemotherapy in Rectal cancer: a pooled analysis of 3085 patients from 7 randomized trials. Radiotherapy and Oncology 2021; 154: 154–60. doi: 10.1016/j.radonc.2020.09.026 32966845

[72] Perez RO, Habr-Gama A, São Julião GP, Lynn PB, Sabbagh C, Proscurshim I, et al . Predicting complete response to Neoadjuvant CRT for distal Rectal cancer using sequential PET/CT imaging. Tech Coloproctol 2014; 18: 699–708. doi: 10.1007/s10151-013-1113-9 24509716

[73] Van den Begin R, Kleijnen J-P, Engels B, Philippens M, van Asselen B, Raaymakers B, et al . Tumor volume regression during preoperative Chemoradiotherapy for Rectal cancer: a prospective observational study with weekly MRI. Acta Oncologica 2018; 57: 723–27. doi: 10.1080/0284186X.2017.1400689 29157069

[74] Fernandez LM, Figueiredo NL, Habr-Gama A, São Julião GP, Vieira P, Vailati • Bruna B., et al . Salvage surgery with organ preservation for patients with local regrowth after watch and wait: is it still possible Dis Colon Rectum 2020; 63: 1053–62. doi: 10.1097/DCR.0000000000001707 32692070

[75] Haak HE, Žmuc J, Lambregts DMJ, Beets‐Tan RGH, Melenhorst J, Beets GL, et al . The evaluation of follow-up strategies of watch-and-wait patients with a complete response after Neoadjuvant therapy in Rectal cancer. Colorectal Dis 2021; 23: 1785–92. doi: 10.1111/codi.15636 33725387

